# Clinical Cases

**Published:** 1899-05

**Authors:** F. S. Davis

**Affiliations:** Quincy, Mass.


### CLINICAL CASES.

Dr. F. S. Davis, Quincy, Mass.

April 12th, 1898.—Case of boy, J. J., æt. 5 years. A strong-looking child, dark complexioned; went to bed well, woke up about 12 o'clock very croupy; very much troubled for breath; would jump up and struggle; did not seem to know what he was doing.

After the struggle had wakened him up fully, would take a drink of water; would quiet down, and while not breathing easily would drop off to sleep; would perspire profusely during his exertions for breath. Pulse would be full, irregular, and so quick as not to be counted.

The only symptom decisive of a remedy was the aggravation after every sleep of any length; if more frequently awakened, was not so bad; if he slept quite a while was certain to have a severe time when he did waken. Lach. 1M., one dose dry.

Attacks became at once milder, and in a few hours he slept quietly. Was well the next day; was over his trouble. This same child had a very severe attack of croup two years or more ago, and in consultation Dr. S. A. Kimball, of Boston, gave it as his opinion that it was of a membraneous character. Kali-bi. was at last given, and relieved, but his recovery was somewhat slow, for he was very weak.

April 2d, 1898.—Mr. A. W. L., minister, æt. 44, a dark-complexioned, well-built man, presented the following symptoms, which so accurately corresponded to those of Zincum that I did not hesitate to give it, and the improvement was very marked both in his mental state and appearance, and in his ability to work. He had been much overworked and complained of feeling all played out—exhausted mentally and physically. As he expressed it, "I am a complete wreck," "I can't control myself," "Can't keep my feet still." Forgetful, cannot keep mind on his work or follow out a line of thought. Feels anxious and apprehensive regarding things that never had been of trouble to him. Sensitive to other's talking; cannot endure his little girl's talking or play; feels so vexed, wants to drive every one away. Talking distresses him. Easily irritated and startled; wishes he could vent his spite on some one; vexed with himself and every one; disinclined to work; soreness in abdomen and side under short ribs; a fullness as if had eaten over much. Nose feels sore; cannot help picking it; bores nose with fingers if he cannot control himself. Lips dry; picks at them more from nervousness. Several years ago I cured him of a deep crack of lower lip with Nat-mur. (Zincum also has cracked lips.)

The greatest trouble he had, so far as preventing work was concerned, was a weak, husky, nasal voice; could not get his voice out; could not get chest tones. This was very much changed for the better by Zincum, so that his voice was natural and of volume such as he never had before, and it had entirely lost its nasal sound, and the throat clear; no huskiness which had existed so long. The nervous strain of talking was not felt, and, as he expressed it, "I am a new man."

All this was accomplished by two doses of Zincum, CM., the last given April 14th, on account of a setback which was caused by over-exertion mentally and physically bringing on a nervous weakness, which he located at the navel, extending through to the back. Arms felt as if not under his control; much lack of confidence in himself, and no inclination to work. Mental or physical exertion, even a very little, greatly exhausted him. Legs felt heavy, and as if he was dragging them along after him. These conditions did not affect his voice, which was as good as new all the time—and is so to-day—and he is gaining fast and feeling like a new man.

---

